# Young Male With End-Stage Renal Disease Due to Primary Hyperoxaluria Type 2: A Rare Presentation

**DOI:** 10.7759/cureus.46555

**Published:** 2023-10-05

**Authors:** Pranjal Kashiv, Shubham Dubey, Kapil N Sejpal, Sunny Malde, Prasad Gurjar, Amit Pasari, Manish Balwani

**Affiliations:** 1 Nephrology, Jawaharlal Nehru Medical College, Datta Meghe Institute of Higher Education & Research, Wardha, IND

**Keywords:** primary hyperoxaluria type 2 (ph2), genetic testing, dialysis, end-stage renal disease, nephrolithiasis

## Abstract

Primary hyperoxaluria type 2 (PH2) is a rare genetic disorder characterized by excessive oxalate production due to glyoxylate metabolism alterations. This case report presents a 26-year-old male with PH2 who experienced recurrent nephrolithiasis since childhood, leading to end-stage renal disease (ESRD). The patient's history prompted genetic testing, which revealed a heterozygous missense variant in the *GRHPR* gene, confirming PH2. Early genetic diagnosis is crucial for preventing ESRD and planning effective treatments. Patients with PH2 require intensive hemodialysis and may benefit from kidney transplantation. However, even after transplantation, ongoing preventive measures are essential due to the risk of hyperoxaluria-related graft damage. This case highlights the importance of early detection and genetic testing in managing PH2 to delay ESRD and improve patient outcomes.

## Introduction

Primary hyperoxaluria (PH) is a rare autosomal recessive disorder characterized by excessive oxalate production due to alterations in glyoxylate metabolism [1]. There are three types of PH: PH1, PH2, and PH3. PH2 results from the absence of glyoxylate reductase/hydroxy pyruvate reductase (GRHPR), which normally converts glyoxylate to glycolate [2], leading to increased urinary excretion of oxalate and L-glyceric acid. PH1 is the most common type, accounting for 80% of PH cases, with a prevalence of 1 case in 1-3 million worldwide [3]. PH2 accounts for approximately 10% of cases, with a prevalence of 1 chance in 4-5 million worldwide [4,5].

PH can lead to recurrent nephrolithiasis and, less commonly, nephrocalcinosis [6]. As kidney function declines, oxalate deposition accelerates, potentially causing systemic oxalosis, a life-threatening condition that affects multiple organs. The median age of end-stage renal disease (ESRD) in PH2 is 40 years, with reported symptom onset typically occurring between 3.2 and 7.4 years of age. Approximately one-quarter to one-third of PH2 patients develop ESRD, while about one-third maintain normal renal function [7].

In this case report, we describe a patient with PH2 who developed ESRD due to persistent nephrolithiasis since childhood. We emphasize the importance of early detection of this genetic disorder for implementing preventive strategies against recurrent nephrolithiasis and delaying ESRD.

## Case presentation

**Figure 1:**
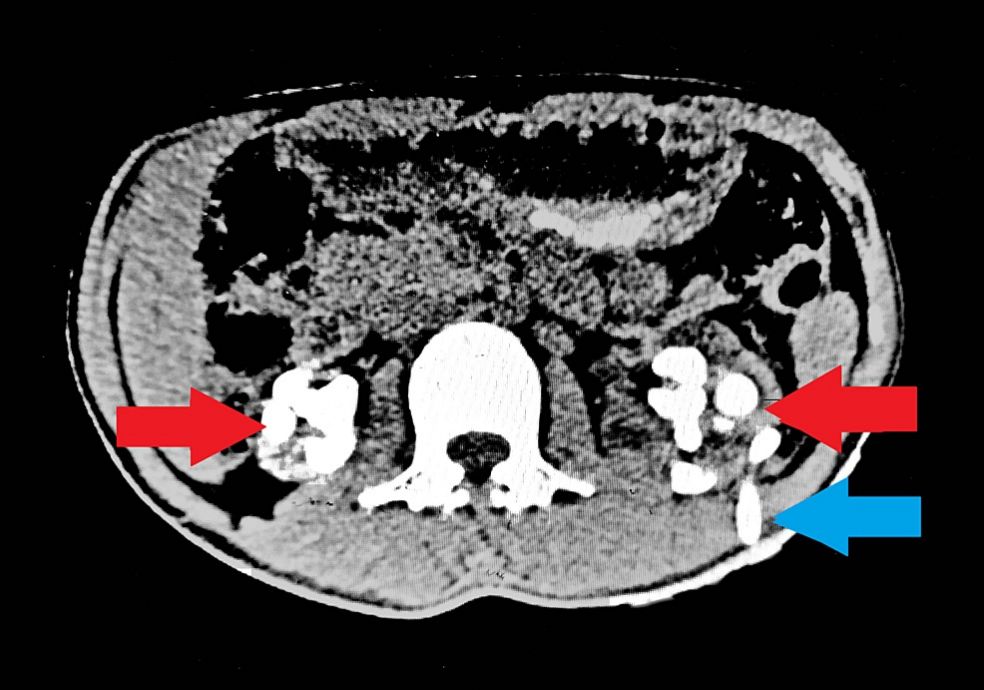
NCCT KUB image NCCT KUB showing bilateral staghorn (red arrow) calculi with left PCN (blue arrow). NCCT KUB: Non-contrast computerized tomography scan of the kidneys, ureter, and bladder; PCN: Percutaneous nephrostomy

**Figure 2:**
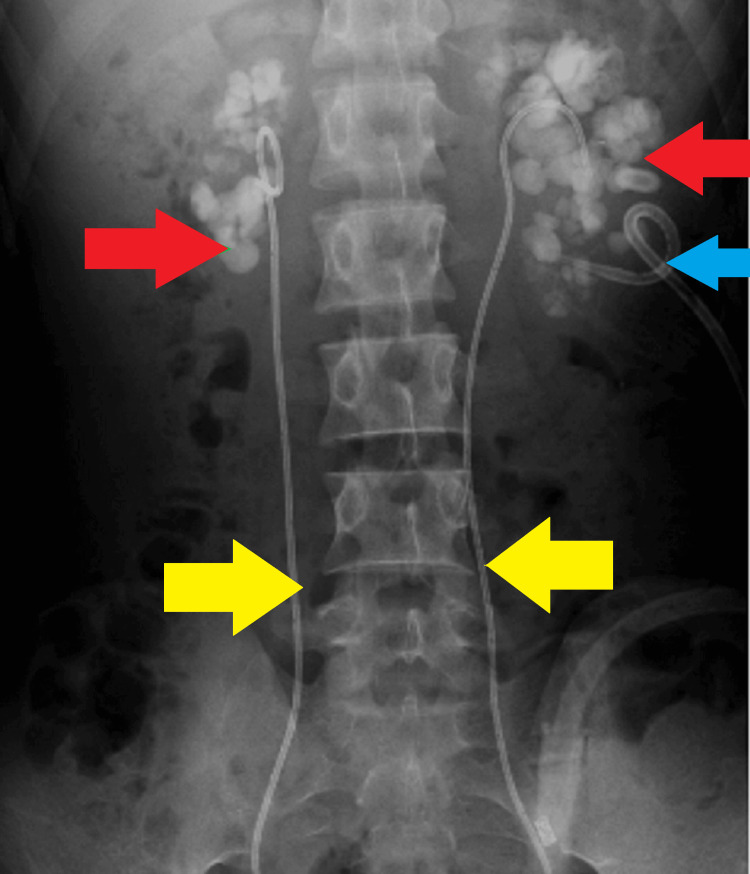
X-ray KUB X-ray KUB showing bilateral staghorn calculi (red arrow) with bilateral DJ stenting (yellow arrow) and left PCN (blue arrow). KUB: Kidneys, ureter, and bladder; PCN: Percutaneous nephrostomy

We present the case of a 26-year-old male admitted to another hospital with reduced urine output, vomiting, and anorexia. An evaluation revealed bilateral staghorn calculi, bilateral hydroureteronephrosis, and renal cortical thinning. He underwent left-sided percutaneous nephrostomy (PCN) insertion (Figure 1) but continued to experience severe uremia, leading to hemodialysis and subsequent double-J (DJ) stent placement (Figure 2). However, urine output did not improve after DJ stenting, necessitating ongoing hemodialysis and left distal arteriovenous (AV) fistula creation at the same hospital.

The patient was referred to our hospital for maintenance hemodialysis. His history included recurrent nephrolithiasis since childhood, prompting consideration of a genetic cause. The patient consented to genetic testing, given this history. Since he was anuric, a 24-hour urine collection for metabolic testing was not possible, prompting direct genetic analysis. The analysis revealed a heterozygous missense variant in exon six of the GRHPR gene consistent with PH2. Additional evaluations for oxalate deposits in other organs, including ECG, 2D Echo, retinal screening, and slit lamp examination, were all normal.

## Discussion

PH2 is considered a rare genetic disorder, but it is often underdiagnosed. Our case highlights the frequent misdiagnosis or disregard of PH2 over the years [7]. Early genetic testing is essential for patients with confirmed PH2 to delay ESRD progression [8]. The GRHPR gene, located on chromosome 9p11, encodes a 36 kDa protein, and approximately 40 mutations have been identified, including deletions, insertions, misspellings, and nonsense mutations [5,9]. Patients with PH2 exhibit significantly elevated urinary oxalate excretion, ranging from 135-270 mg/1.73 m^2^ per day, compared to normal levels of less than 45 mg/1.73 m^2^ per day [10]. PH2 patients often present with elevated L-glyceric acid levels (>28 mmol/mol creatinine) [5,11].

Once diagnosed, preventive measures aim to reduce renal oxalate deposition and delay chronic kidney disease (CKD) progression. Strategies include increased water intake, pyridoxine therapy, crystallization inhibitors, and vitamin E therapy. Dietary oxalate intake should be limited, avoiding foods high in oxalates such as rhubarb, chocolate, spinach, and tea [12].

The diagnosis of PH2 is critical as it impacts hemodialysis (HD) treatment. PH2 patients require frequent and extended HD sessions, typically lasting 4-5 hours, 4-5 times a week. The majority also require both HD and peritoneal dialysis (PD) [13]. However, conventional HD and PD offer limited oxalate clearance (950-1440 micromol/day), significantly lower than the daily oxalate production (3500-7500 micromoles) in PH2 patients [14,15]. Plasma oxalate concentrations decrease by 60-80% after HD but return to 80% and 95% of pre-dialysis levels after 24 and 48 hours, respectively. This delay in plasma oxalate reduction can lead to systemic oxalate intoxication despite maintenance dialysis, as plasma oxalate often exceeds the 30 micromol/L supersaturation threshold between treatments. Intensive HD before kidney transplantation can help reduce plasma oxalate levels and prevent subsequent oxalate deposition and graft tissue damage [16].

For PH2 patients progressing to ESRD, isolated kidney transplantation is often considered a suitable treatment approach [1,17]. Although combined liver/kidney transplants have shown some benefits, more research is needed to determine their efficacy in PH2 patients [7,18].

Even after kidney transplantation, PH2 patients may experience hyperoxaluria due to the mobilization of tissue oxalate stores. High fluid intake and crystallization inhibitors are recommended to protect the transplanted kidney as long as urinary oxalate remains elevated. However, graft loss can still occur due to oxalate deposition from newly transplanted kidneys and mobilized oxalate.

## Conclusions

Metabolically active stones, recurrent nephrolithiasis, family history of nephrolithiasis, nephrolithiasis in young individuals, and nephrolithiasis with nephrocalcinosis warrant stone analysis and evaluation. In cases of end-stage renal disease, isolated kidney transplantation is the preferred treatment for PH2. Early genetic testing is crucial for diagnosis and can prevent or delay renal failure, leading to successful transplantation without recurrent oxalosis.
